# Antibacterial and Hemocompatible pH-Responsive Hydrogel for Skin Wound Healing Application: In Vitro Drug Release

**DOI:** 10.3390/polym13213703

**Published:** 2021-10-27

**Authors:** Muhammad Umar Aslam Khan, Saiful Izwan Abd Razaq, Hassan Mehboob, Sarish Rehman, Wafa Shamsan Al-Arjan, Rashid Amin

**Affiliations:** 1 BioInspired Device and Tissue Engineering Research Group, School of Biomedical Engineering and Health Sciences, Faculty of Engineering, Universiti Teknologi Malaysia, Skudai 81300, Johor, Malaysia; 2 Institute of Personalized Medicine, School of Biomedical Engineering, Med-X Research Institute, Shanghai Jiao Tong University (SJTU),1954 Huashan Road, Shanghai 200030, China; umar-786@sjtu.edu.cn; 3 Nanosciences and Technology Department (NS & TD), National Center for Physics, Quaid-i-Azam University Campus, Islamabad 44000, Pakistan; 4 Centre for Advanced Composite Materials Universiti Teknologi Malaysia Skudai, Johor Bahru 81310, Johor, Malaysia; 5 Department of Engineering Management, College of Engineering, Prince Sultan University, Rafha Street, P.O. Box 66833, Riyadh 11586, Saudi Arabia; hmehboob@psu.edu.sa; 6 Chemistry Department, McGill University, 801 Sherbrooke St. W, Montreal, QC H3A0G4, Canada; sarishrehman1@qmail.com; 7 Department of Chemistry, College of Science, King Faisal University, Al-Ahsa 31982, Saudi Arabia; walarjan@kfu.edu.sa; 8 Department of Biology, College of Sciences, University of Hafr Al Batin, Hafar Al-Batin 39524, Saudi Arabia; rashidamin75@googlemail.com

**Keywords:** antibacterial, biomaterials, biopolymers, controlled drug release, hemocompatibility, kinetics studies, wound dressing

## Abstract

The treatment of successive skin wounds necessitates meticulous medical procedures. In the care and treatment of skin wounds, hydrogels produced from natural polymers with controlled drug release play a crucial role. Arabinoxylan is a well-known and widely available biological macromolecule. We produced various formulations of blended composite hydrogels (BCHs) from arabinoxylan (ARX), carrageenan (CG), and reduced graphene oxide (rGO) using and cross-linked them with an optimal amount of tetraethyl orthosilicate (TEOS). The structural, morphological, and mechanical behavior of the BCHs samples were determined using Fourier-transform infrared spectroscopy (FT-IR), Scanning electron microscope (SEM), mechanical testing, and wetting, respectively. The swelling and degradation assays were performed in phosphate-buffered saline (PBS) solution and aqueous media. Maximum swelling was observed at pH 7 and the least swelling in basic pH regions. All composite hydrogels were found to be hemocompatible. In vitro, silver sulfadiazine release profile in PBS solution was analyzed via the Franz diffusion method, and maximum drug release (87.9%) was observed in 48 h. The drug release kinetics was studied against different mathematical models (zero-order, first-order, Higuchi, Hixson–Crowell, Korsmeyer–Peppas, and Baker–Lonsdale models) and compared their regression coefficient (R^2^) values. It was observed that drug release follows the Baker–Lonsdale model, as it has the highest value (0.989) of R^2^. Hence, the obtained results indicated that, due to optimized swelling, wetting, and degradation, the blended composite hydrogel BCH-3 could be an essential wound dressing biomaterial for sustained drug release for skin wound care and treatment.

## 1. Introduction

Skin is the largest human organ that is 10% of body mass. It protects our body from bacterial, viral infections, and also from environmental factors. In addition, it also controls physiological protective mechanisms, sensing, temperature regulation, fluid maintenance, and the immune system. Skin injuries are one of the most prevalent medical problems in the history of humanity [1,2]. Therefore, the development of new dressing materials was an essential concern in modern medical technology. Hydrogels with high moisture and bioactivity are considered promising candidates for potential impact among these modern dressing materials formulated [3]. First, by offering a rough surface and an appropriate swelling ratio, the hydrogel matrix can enable the presence of oxygen, absorb the exudates, and maintain moist restorative surroundings for wound healing [4]. Second, hydrogel adhesives prevent bacterial growth and encourage gaseous exchange that inhibits anaerobic bacteria proliferation. Third, the antibacterial properties of conventional dressing are also enriched with capsulated antibiotics into a polymeric network of hydrogels [5]. Therefore, wound dressings with inherent antibacterial properties are more appealing since they offer more potent antibacterial activity and biocompatibility. Finally, modern wound dressings are more advanced and different from conventional wound dressings due to loaded bioactive molecules with controlled release and targeted drug delivery [6]. Curcumin, which has powerful modulative antibacterial and biocompatible effects for wound healing, is one of the released drug molecules that play essential roles in wound healing. Fourth, the physiological environment of damaged skin is slightly acidic. Consequently, pH-responsive wound dressing based on hydrogel, which could smartly release drugs, was more effective for the actual needs to heal wound quickly [7].

Hydrogels consist of a three-dimensional hydrophilic polymeric network with insoluble properties. Hydrogels can absorb fluids thousands of times of their original weight until equilibrium. These are usually used for dry or moderately draining wounds, encouraging autolytic debriding in fibrotic wounds and granulation of wounds [8]. Completely swollen hydrogels have a broad spectrum of essential properties that help accelerate wound healing in living tissues, such as elastic and low interfacial tension. The low contact angle between the hydrogel surface and body fluid morphology will minimize protein absorption and cell adherence, facilitating cell proliferation, differentiation, and migration [9]. Biodegradable hydrogels with controlled drug release kill pathogens and provide a microenvironment that promotes quick wound healing [10]. The polymeric hydrogels are similar to the natural extracellular matrix (ECM) related to their structural and mechanical features.

Arabinoxylan is a natural biological macromolecule polymer and is abundantly available in nature. Easy availability and economic approaches make arabinoxylan a potential biopolymer with several potential applications in biomedical sciences [11,12]. Arabinoxylan has gained considerable attention among all biopolymers in biomedical sciences due to its biocompatibility, biodegradability, film formation, antimicrobial and antioxidant properties [13,14]. Several studies show that arabinoxylan-based materials are antibacterial, antitumor, analgesic, hemostatic, nontoxic, highly biocompatible, and antigenic biomaterial [15]. Membranes/films formed from biopolymers have poor mechanical properties controlled through several strategies [16]. The physicochemical characteristics (such as hydrophilic, swelling, biodegradation, and net charge) allow arabinoxylan, an effective biological macromolecule, to the controlled delivery of active ingredients (drugs, growth factors, stem cells, and peptides, etc.). Hydrogel wound dressings based on arabinoxylan with various compositions can facilitate wound recovery and mitigate undesirable wound healing factors. These optimized properties of hydrogels support the absorption of wound exudate, provide required hydration, and enable water take-up to shield the wound from environmental and pathogenic effects [17]. The pH of any wound does not remain the same, and it is changed until its complete healing. This is because of wound exudate, waste and degraded hydrogel, and ions from biofluid and environmental factors. Therefore, these factors are involved in the change in pH, and during the change in pH, the hydrogel behaves differently according to pH level. Hence, it is important to determine the swelling behavior of hydrogel at different pH levels [18].

This research provides information on novel compositions of blended composite hydrogels for skin wound care and treatment. Arabinoxylan (ARX), carrageenan (CG), and reduced graphene oxide (rGO) were cross-linked via tetraethylorthosilicate (TEOS). To the best of our knowledge, these formulations have never been reported before elsewhere. FT-IR, SEM, water contact angle, mechanical testing, in vitro degradation, and swelling analysis were investigated to observe these hydrogels’ surface morphology, hydrophilicity, and pH sensitivity. In addition, in vitro drug (silver sulfadiazine) release was evaluated via Franz transdermal diffusion method. The kinetic studies of silver sulfadiazine were analyzed through different models (zero-order, first-order, Higuchi, Korsmeyer–Peppas, Hixson–Crowell, and Baker–Lonsdale models). The prepared BCHs might be potential biomaterial for the care and treatment of skin wound healing with improved fluid retaining properties.

## 2. Materials and Methods

### 2.1. Materials

Plantago Ovata husk was bought from a local market of Johor Bahru, Malaysia, to extract arabinoxylan (ARX) according to the reported method [19]. Carrageenan (CG) (CAS number: 9000-07-1), TEOS (CAS number: 78-10-4), ethanol, rGO (CAS number: 805424-500MG), phosphate-buffered saline (PBS) solution, and hydrochloric acid (HCl) were purchased from Sigma Aldrich, Selangor, Malaysia. These chemicals were analytically graded and used as received.

### 2.2. Fabrication of Hydrogels

**Figure 1:**
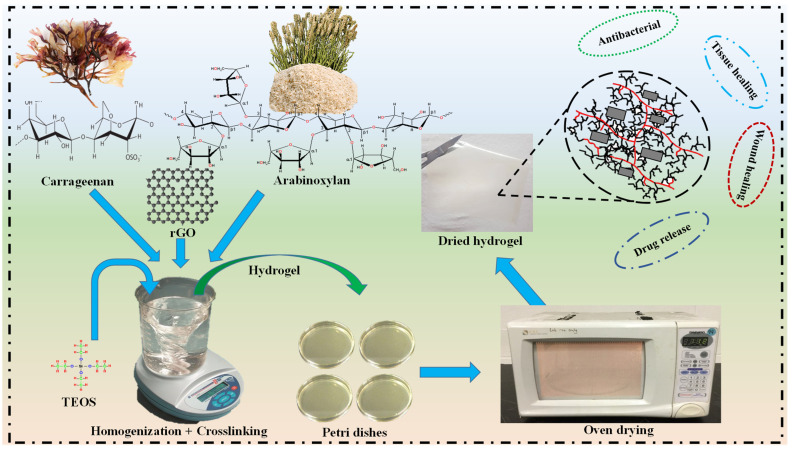
The represents the complete syntheses of composite hydrogel with characterizations and biological activities.

Hydrogels were produced by separately dispersing AR (0.7 g) and CG (0.3 g) into 25 mL deionized water. Both suspensions were mixed to have a homogenized mixture and stirred with different amounts of rGO (0.1, 0.2, 0.3, and 0.4 mg) for 2 h. The homogenized mixture was cross-linked using (TEOS (150 μL)). TEOS was mixed into 4 mL ethanol and dropwise added into AR/rGO/CG mixture. It was stirred for 3 h at 60 °C to prepare with different compositions of blended composite hydrogels. The cross-linked hydrogels were shifted into clean and well-dried Petri glass dishes. These Petri dishes were kept in the oven and dried at 55 °C. The newly developed hydrogels were coded BCH-1, BCH-2, BCH-3, and BCH-4 due to different rGO quantities (0.1, 0.2, 0.3, and 0.4 mg). Figure 1 illustrates the synthesis process of the hydrogel.

## 3. Characterization

### 3.1. Fourier Transform Infrared (FT-IR)

Functional group analysis of well-dried hydrogel samples was conducted using Fourier transform infrared (FT-IR) spectroscopy (Nicolet 5700, Waltham, MA, USA), ranging from 4000 to 400 cm^−1^ 150 routine scans.

### 3.2. Scanning Electron Microscope (SEM)

A scanning electron microscope (SEM) (JSM-6701S, Peabody, MA, USA) was used to analyze the morphology of hydrogel samples. Well-dried hydrogel samples were gold-spurted before morphological analysis.

### 3.3. Wetting Analysis

The water contact angle technique was used to determine the hydrophobicity and hydrophilicity of hydrogels. In addition, the water contact angle system (JY-82, Dingsheng, Chengde, China) was used to assess wetting analysis.

### 3.4. Mechanical Testing

The mechanical properties of blended composite hydrogels were evaluated using the tensile tests. Samples were shaped into strips for tensile testing. The tensile test was conducted at a 10 mm/min speed. The overall length of this dumbbell-shaped specimen is 115 mm, with a narrow section of 33 mm, a gauge length (benchmark) of 25 mm, and a gauge width of 6 mm.

### 3.5. Swelling, Biodegradation, and Water Content

At the different pH levels, the swelling study of hydrogels was conducted. Therefore, hydrogels (BCH-1, BCH-2, BCH-3, and BCH-4) were cut in a square shape, and initial weight (*W_i_*) was determined (50 mg). These hydrogels were soaked into a beaker (100 mL) of analogous pH media at room temperature. Then, hydrogels were taken out from the media after a fixed time and weighed (*W_f_*) at room temperature. The excess surface solvent was removed carefully from tissue paper and observed weight of the swollen hydrogels. The percentage of swelling was determined by Equation (1).

(1) $$Swelling\;\left(\%\right)=\frac{W_{f}-W_{i}}{W_{i}}\times 100$$

The in vitro biodegradation was investigated by immersing hydrogels into PBS solution (pH 7.4) and incubating at 37 °C under 5% CO_2_ for 1, 2, 3, 5, and 7 days. Hydrogels were cut into a square shape and weighed (35 mg) (BCH-1, BCH-2, BCH-3, and BCH-4). The weight of hydrogels was calculated before and after degradation under sterile conditions to protect from bacterial and fungal contamination. The weight loss of all samples of hydrogel was calculated by Equation (2).

(2) $$Weight\;loss\;\left(\%\right)=\frac{W_{o}-W_{i}}{W_{o}}\times 100$$

Specifically, the following procedure was used to determine the water content of each hydrogel. First, these mixed composite hydrogels were weighed (*W_o_*) with a 20 mm radius and 4 mm thickness. The weight of the blended composite hydrogels was again weighed (*W_i_*) after drying for 24 h. Finally, we measured the water content (M) from Equation (3) as follows:

(3) $$Water\;content\;\left(\%\right)=\frac{W_{o}-W_{1}}{W_{o}}\times 100$$

### 3.6. In Vitro Studies

#### 3.6.1. Antibacterial Activity

Valgas et al. reported on the antibacterial activities of hydrogels against severe disease-causing bacteria using the disc diffusion method [20]. These bacterial strains Pseudomonas aeruginosa and Escherichia coli (*P. aeruginosa* and *E. coli*) (Gram negative (-ive)) and Staphylococcus aureus (*S. aureus*) (Gram positive (+ive)) were supplied by ATCC (Manassa, VA, USA). The bacterial strains were aerobically cultivated utilizing Müller–Hinton broth (MHB) and tryptone soy agar (TSA), Sigma Aldrich, Selangor, Malaysia. These were grown overnight over Luria–Bertani (LB) broth, forming a turbid suspension in a sterile saline solution (0.85%). The Müller–Hinton agar (MHA) melted 20 mL was poured into the Petri dish and let to be solidified. The bacterial strain was spread over the MHA plate on solidification by a glass spreader. Then, 85 µL of hydrogels were dropped in each Petri dish well and incubated for 24 h at 37 °C with the help of a micropipette. The concentration was altered at 530 nm through a spectrophotometer to obtain an average concentration for McFarland (0.5 − 25 × 103). The bacterial zone of the inhibitions was evaluated (in mm), and the Clinical Laboratory Standards Institute (CLSI) disc diffusion breakpoints were used to interpret these areas.

#### 3.6.2. Hemocompatibility Assay

The hemocompatibility assay of composite hydrogels was evaluated against healthy human blood collected from the trauma center, Jinan Central Hospital, Shandong Province, China. The conducted study was approved by the Ethics Committee as obtained under UTM/2016/KHAIRUL NADWA/28-JAN./729-FEB-2016-JAN-2019. The hemocompatibility assay was studied for all the composite hydrogels by the well-reported method by Li et al. with slight modifications [21]. Fresh red blood cells were centrifuged at 500× *g* for 5 min before being washed repeatedly in a PBS solution (pH 7.4). Composite hydrogel samples (100 g/mL) were prepared at 37 °C in PBS (pH 7.4) solution, and blood samples were diluted in PBS solution. For 1 h, these blood samples were incubated at 37 °C. The controls (+ive and −ive) were incubated at 37 °C in Triton (1%) and PBS media and centrifuged at 1500× *g* for 5 min. The supernatant absorbance was recorded at λ = 540 nm to observe hemoglobin releasing by a UV–vis spectrophotometer, (UV-Visible (HACH D500), Loveland, CO, USA), and hemolysis% was calculated by Equation (4).

(4) $$Hemolysis\left(\%\right)=\frac{Abs_{S}-Abs_{NC}}{Abs_{PC}-Abs_{NC}}\times 100$$

#### 3.6.3. Drug Loading and Drug Delivery via Franz Diffusion Method

Silver sulfadiazine (20 mg) was accurately weighed and dissolved in ethanol (5 mL). The drug was then added dropwise into the AR and CG mixture and stirred continuously for 2 h to achieve a homogenized mixture. After 2 h, TEOS (200 μL) was added with constant stirring at 60 °C. According to Saiful et al., a drug-loaded hydrogel was prepared. Drug delivery was studied using the Franz diffusion method using PBS buffer solution (7.4 pH) at 37 °C [22]. Every 10 min, 2 mL of the sample was taken and measured using a double beam UV–vis spectrophotometer. The PBS buffer solution was used as a reference standard, and graph calibration determined drug release.

#### 3.6.4. Release Kinetics of Silver Sulphadiazine

The release of in vitro transdermal medication (silver sulphadiazine) in PBS solution was described using mathematical models Equations (4)–(9). In addition, the drug release kinetic studies [23,24,25,26] for sliver sulfadiazine drug were studied as a function of time against various models (zero-order, first-order, Higuchi, Korsmeyer–Peppas, Hixson–Crowell, and Baker–Lonsdale models).

(5) $$\mathrm{Zero}-\mathrm{order}\;\;\;\;M_{t}=M_{o}+K_{o}t$$

(6) $$\mathrm{First}-\mathrm{order}\;\;\;\;logC_{o}-\frac{kt}{2.303}$$

(7) $$\mathrm{Higuchi}\;\mathrm{model}\;\;\;\;ft=Q=K_{H}\times t^{1/2}$$

(8) $$\mathrm{Hixson}–\mathrm{Crowell}\;\mathrm{model}\;\;\;\;W^{1/3}-W^{1/3}=kt$$

(9) $$\mathrm{Korsmeyer}–\mathrm{Peppas}\;\mathrm{model}\;\;\;\;ln\frac{M_{t}}{M_{o}}=n\;lnt+lnK$$

(10) $$\mathrm{Baker}–\mathrm{Lonsdale}\;\mathrm{model}\;\;\;\;F_{t}=\frac{2}{3}\left[1-{\left(1-\frac{M_{t}}{M_{o}}\right)}^{\frac{2}{3}}\right]\frac{M_{t}}{M_{o}}=K{\left(t\right)}^{0.5}$$

where *M_t_* = drug release amount at time *t*; *KH*, *K*, and *K_o_* are constants.

### 3.7. Statistical Analysis

The data were evaluated in triplicate and presented as mean standard error (SE) and shown in Y-error bars. The data were statistically analyzed with the help of statistical software (IBM, SPSS Statistics 21, SPSS Inc., New York, NY, USA). (*p* < 0.05; size of sample *n* = 3)

## 4. Results and Discussions

### 4.1. FT-IR Analysis

**Figure 2:**
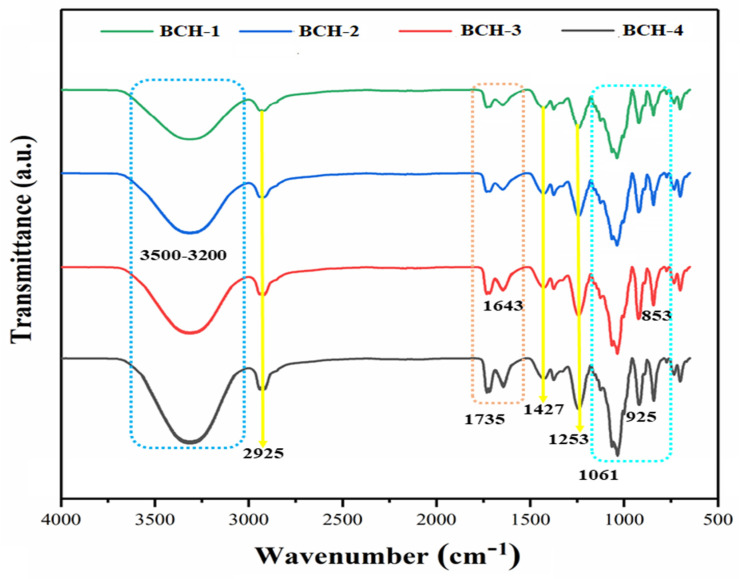
FT-IR spectrum of all samples of biocomposite hydrogel with different functional groups exhibiting successful cross-linking.

FT-IR spectral profile of blended composite hydrogels is shown in Figure 2 to investigate the behavior of functional groups of all elements and their reaction possibilities. The more substantial broadband absorbance 3500–3200 cm^−1^ explains the intermolecular and intramolecular hydrogen bonding of arabinoxylan and carrageenan. The peak at 2925 cm^−1^ was attributed to –CH2 stretching vibration [11]. The adsorption peaks at 1061 and 768 cm^−1^ were due to asymmetric vibration of Si–O–Si; peaks at 925 and 1630 cm^−1^ were attributed to Si–OH, and peak at 1267 cm^−1^ was due to Si–C. These functional Si–O–Si, Si–OH, and Si–C confirmed successful TEOS cross-linking [27]. The vibration peaks occurring at 1725 and 1643 cm^−1^ were attributed to the carbonyl groups (C=O) and carboxylic acid groups (COOH). The increasing amount of rGO led to increased intensity, and the adsorption bands at 1469, 1349, and 1253 cm^−1^ were reduced, showing graphene oxide fundamental characteristic peaks [15,28]. The absorption vibrations bands at 1147 and 853 cm^−1^ are characteristic peaks of saccharine structure and pyranose ring that confirm arabinoxylan and carrageenan, respectively. As a result, all possible vibrational peaks in the FT-IR spectrum were identified, and peak analysis verified that all elements were present, confirming the successful preparation of blended composite hydrogels.

### 4.2. SEM Analysis

**Figure 3:**
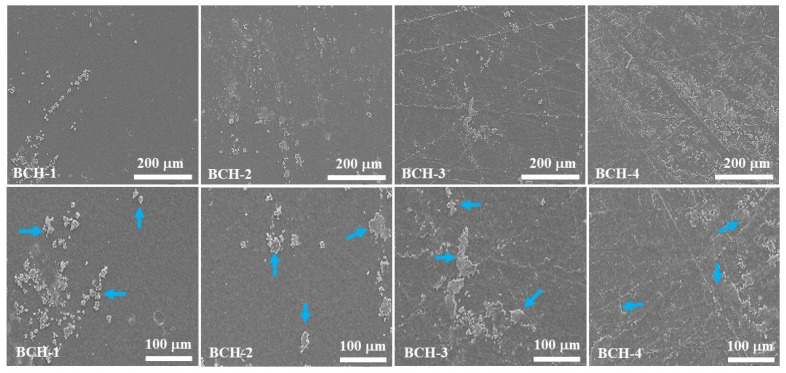
SEM micrographs of all samples of biocomposite hydrogels and in the first row, the SEM micrographs were captured at magnifications (200 at 100 µm). The blue arrows in the second-row present flakes of rGO, causing rough surface morphology.

The surface morphology of blended composite hydrogels was examined at various magnifications to understand their behavior better, as shown in Figure 3. Due to the aggregation of rGO over the surface, the surface morphology of these composite hydrogels was smooth with nanoflakes, as shown by blue arrows. Nanoflakes formed on the surface of blended composite hydrogels as the rGO content increased, contributing significantly to changes in surface morphology. For example, BCH-1 contained fewer nanoflakes, whereas BCH-4 contained the most nanoflakes, which changed the smooth surface to the rough surface. Figure 3 shows SEM surface micrographs of blended composite hydrogels at various magnifications. According to SEM micrographs, the composite hydrogels had a very smooth, uniform, and nonporous surface morphology.

Nonetheless, the introduction of rGO in blended composite hydrogels with various contents of 0.1 to 0.4 mg resulted in microscopic rGO aggregates on the surface. The number of these nanoflakes increased as the rGO content on the surface increased. Furthermore, nanoflakes were found on the surface of mixed composite hydrogels in significant numbers. Cell adhesion, differentiation, and migration are aided by rough surface morphology [29]. rGO nanoflakes were in different agglomerations compared with smooth surface area and rough morphology (Figure 3). While excessive amounts of rGO in blended composite hydrogels can obstruct the proper preparation of a polymeric matrix with miscibility, increasing the amount of rGO provides more cross-linking. The homogeneous, hydrophobic, or miscibility behavior of the biopolymeric matrix and rGO components could also be attributed to morphological differences. The introduction of high rGO material explained this behavior, contributing to developing a high amorphous form and structural toughness. As a result, as the amount of rGO increased, the nanoflakes phase increased, resulting in rough surfaces due to the high rGO content.

### 4.3. Wetting Analysis

**Figure 4:**
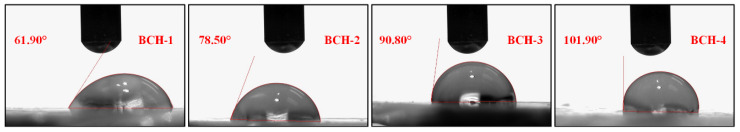
The measurement of water contact angles at different time intervals to determine the hydrophilicity of hydrophobicity of hydrogels with an increasing amount of rGO and time (t = 5 s).

Wetting analyses are used to determine whether a blended composite hydrogel is hydrophilic or hydrophobic, which aids in understanding surface cell activity during biological activities [30]. We examined the wetting behavior of mixed composite hydrogels at different time intervals (5 s) and observed that the hydrophobic behavior changed to hydrophilic over time. At zero second, BCH-1 = 61.90°, BCH-2 = 78.50°, BCH-3 = 90.80°, and BCH-4 = 101.90° contact angles were measured for all composite hydrogels (BCH-1 = 61.90°, BCH-2 = 78.50°, BCH-3 = 90.80°, BCH-4 = 101.90°). As a result of increasing rGO, the water contact angle increased, and the wetting behavior shifted from hydrophilicity to hydrophobicity, as shown in Figure 4. As a result of increasing rGO, the water contact angle increased, and the wetting behavior shifted from hydrophilicity to hydrophobicity, as shown in Figure 4. The hydrophobicity of BCH-2 increased slightly as the amount of rGO increased.

Similarly, BCH-4 hydrophobicity is higher than all other hydrogels due to the higher rGO content, promoting efficient cross-linking between AR and CG due to weak attraction forces [30,31]. These findings suggest that a variable amount of rGO can obtain the hydrophilic/hydrophobic characteristics in these blended composite hydrogels. Wetting phenomena are critical in surface morphology and polymeric matrix, resulting in significant cell adhesion due to water solubility and hydrogen bonding. As a result of cross-linking, mixed composite hydrogels with specific wetting behavior advantageous for wound healing were developed.

### 4.4. Mechanical Testing

**Figure 5:**
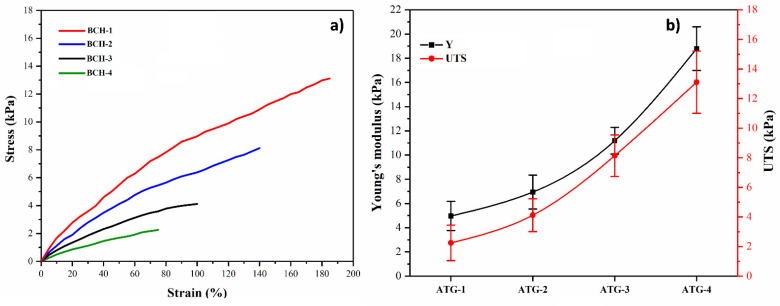
The mechanical behavior of all hydrogels was studied: (**a**) tensile stress–strain curve and (**b**) relationship between Young’s modulus and ultimate tensile strength.

Material healing skin wounds require mechanical properties that are similar to natural skin. It can help with excellent adhesion by an external force during skin tissue deformation by retaining structural integrity. The stress–strain curves were obtained from the tensile tests of blended composite hydrogels (Figure 5a). Young’s modulus and ultimate tensile strengths were calculated from the stress–strain plots shown in Figure 5b. These blended composite hydrogels had a higher Young’s modulus (kPa) than human skin (0.42 to 0.85 MPa) and had better mechanical properties [32]. As the rGO content (0.1, 0.2, 0.3, and 0.4 mg) increased from BCH-1 to BCH-4, the break elongation of the blended composite hydrogels increased. The ultimate tensile strain strength was the highest in BCH-4, with the highest rGO content (about 48.71%), and tensile strength increased from 2.33 to 11.13 kPa by increasing rGO, which is because rGO is the only factor that leads to an excessive number of active sites. Due to the variety of functional groups and the high cross-linking density, the hydrogel was hard rather than soft—reduced cross-linking density resulted in poor mechanical behavior. Increased cross-linking density, on the other hand, improved the mechanical properties of the blended composite hydrogels. As a result, these blended composite hydrogels had mechanical properties comparable to or better than human skin, allowing them to withstand external forces without fracturing and preventing tissue damage.

### 4.5. Swelling Analysis

**Figure 6:**
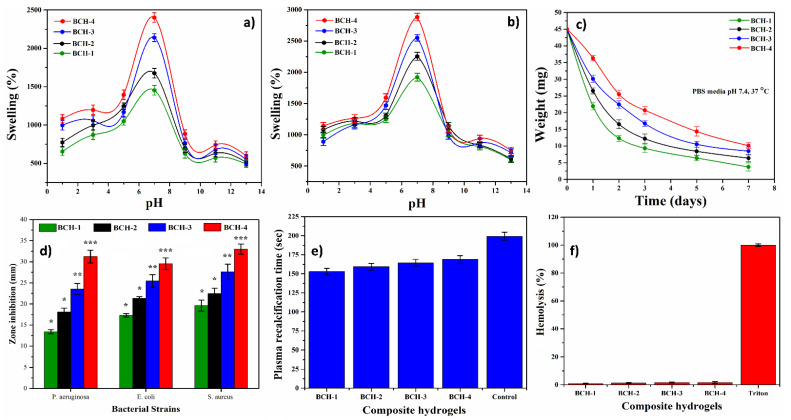
Swelling analysis of hydrogels: (**a**) buffer, (**b**) nonbuffer, (**c**) degradation of hydrogels under in vitro conditions, (**d**) antibacterial activities against different bacterial strains, (**e**) plasma recalcification, and (**f**) hemolysis percentage. (* *p* < 0.05, ** *p* < 0.01 and *** *p* < 0.001).

Swelling is a characteristic of hydrogels that occurs when a solvent penetrates into the void space of the polymeric chain network, causing hydrogels to swell. External stimuli (such as pH, ionic strength, and temperature, etc.) can significantly affect swelling behavior due to its large surface areas of hydrogels, which generate high possibilities of biofluid exchange with the body. Composite hydrogels respond faster to present swelling ability than traditional hydrogels [33]. Hydrogels have the ability to hold a considerable amount of water inside of their structures without being degraded into media. The quantity of water in the hydrogel, which is typically in a swollen state, is determined by the nature of the polymer system. Hydrogels have hydrophilic functional groups with a higher affinity to the polymeric backbone, which allows them to hold water in their structure. In response to changes in pH, hydrogels can swell and shrink in a reversible manner [34]. Swellability analysis in various pH media was performed to observe the pH response of hydrogels (2 to 12 pH). The maximum swelling of all hydrogels was observed at pH 7 (Figure 6a,b). The swelling was reduced in the neutral to acidic and neutral to basic media. Protonation of the alcohol and carboxylic acid functionals of AR and CG caused a decrease in swelling from neutral to acidic media. As the pH was reduced from 7 to 1, anion–anion repulsion and hydrogel swelling were reduced. The electrostatic repulsion of the alcoholate and carboxylate groups caused the most swelling at pH 7. The ionized form of alcoholate and carboxylate contain these functional groups [35,36]. Due to carboxyl and alcohol groups over oxidized polysaccharide chains, the swelling of hydrogels was increased from pH 12 to pH 7. Their carboxylic and alcohol groups were turned into carboxylate and alcoholate ions, respectively. These ions increased electrostatic repulsion, allowing more water to be absorbed [37,38]. These findings revealed more swelling at pH values of 7 and a decreasing pattern in acidic and basic pH ranges. The influence of Na+ in solution was the cause of lower swelling in basic pH. It conserved carboxylate anion, which controls the spread of the hydrogel’s twisted chains by delaying anion–anion repulsion efficiency [39].

### 4.6. Biodegradation

To investigate their biodegradation activity against different formulations, in vitro biodegradability of these blended composite hydrogels was conducted in PBS buffer solution in vitro degradation conditions. The primary objective of in vitro biodegradation is to figure out how blended composite hydrogels degrade over time and develop a method to track drug release and other active ingredients. Figure 6c shows that these blended composite hydrogels have different biodegradation behaviors, resulting in different weight loss under similar conditions. It could indicate alkyl links and glycosidic interactions in polysaccharides biopolymers, with the dissociation of these glycosidic bonds contributing to hydrogel biodegradation [40]. The maximum amount of rGO, due to more and more cross-linking density consequent to increasing rGO, resulted in the lowest in vitro biodegradation of BCH-4. BCH-1, on the other hand, had a higher biodegradation rate due to a lower amount of rGO in blended composite hydrogels with lower cross-linking density. AR and CG contain alkyl and glycosidic linkages, and their dissolution has aided in the biodegradation of the blended composite hydrogels [41]. The successful cross-linking of AR, rGO, and CG via TEOS cross-linker to form composite hydrogel networking is evinced by the different biodegradation rates of all hydrogels.

### 4.7. In Vitro Studies

#### 4.7.1. Antimicrobial Activity

Antibacterial activities against bacteria that cause severe wound skin disease—namely, *P. argenosa*, *E. coli* (Gram negative (−ive)), and *S. aureus* (Gram positive (+ive))—were examined, and their activities were measured in terms of zones of inhibition (mm), as shown in Figure 6d. BCH-1 was the least antibacterial among all blended composite hydrogel samples, while BCH-4 was the most antibacterial. The order of antibacterial efficiency is BCH-4 > BCH-3 > BCH-2 > BCH-1, which could be due to hydrophobic behavior that played a significant role in the bacterial killing process. [42]. Due to the complex structure of *E. coli* bacteria, these hydrogels were more active against *P. argenosa* (Gram negative) and *S. aureus* (Gram positive) bacteria and less active against *E. coli* (Gram negative). These microbes are the most frequently separated from severe wounds, and their colonization necessitates careful management. When tissues are traumatically injured, this can result in a wound infection [43]. We found structural components of tissues (including fascia) that cause virulent *P. aeruginosa* to be produced by scavenging iron ions and affect the host immune system. Antibiotic resistance can develop in these organisms, and they are linked to nosocomial infections [44,45]. Increased rGO quantity inhibited antibacterial properties as nanosheet ruptured bacteria, resulting in significant inhibition zones. The charge transfer caused by multifunctional rGO flakes causes bacterial death and antibacterial activities [46]. The polymeric part of the hydrogels, on the other hand, interacts with the cytoplasmic membrane as a result of strong electrostatic interactions between bacteria’s negative charge and charged hydrogels with multifunctional groups. The interaction of polymeric matrix is capable of hydrogels’ antibacterial effects. A strong coordination interaction compromises the integrity of the bacterial cell wall with carboxyl and carbonyl groups of peptidoglycans. The hydrogel’s polymeric part interacts with bacterial DNA and takes over bacterial control, preventing bacterial growth and, as a result, causing bacterial death [47]. The antibacterial effect of the synergistic effect of polymer and rGO is very strong.

#### 4.7.2. Hemocompatibility and Blood Clotting Time

The wound dressing material must be exceptionally hemocompatible attribute healing processes without causing any hemotoxicity. These composite hydrogels were used in the clinic and will unescapably contact with blood during application. The composite hydrogel binding depends on blood circulation and growth factors involved in wound healing. Thus, performing a hemocompatibility test on composite hydrogels is critical. As a result, in clinical applications, hemocompatibility and clotting time are critical considerations for composite hydrogels. The time it takes for blood to clot and produce thrombin from prothrombin plasma precursor is called coagulation time. The average blood clot time is 2–4 min [48]. The process of turning soluble fibrinogen into insoluble fibrin is known as blood coagulation. The amount of rGO in the blood does not affect clotting or coagulation time [48]. The composite hydrogels triggered coagulation cascades, which resulted in blood clotting. As shown in Figure 6e, adding calcium chloride to citrated blood containing composite hydrogel (BCH-1, BCH-2, BCH-3, and BCH-4) concentrations resulted in a blood clotting time of 152.86–192 s.

During an in vitro hemolysis assay, the composite hydrogels were hemocompatible, compared with the positive control (Triton X100). They only showed minor or no hemolysis, which could be due to the sharp edges of rGO, as shown in Figure 6f. BCH-1, BCH-2, BCH-3, and BCH-4 (100 g/mL) hemolysis levels were 0.9, 1.23, 1.57, and 1.61%, respectively. The positive control (Triton X100), on the other hand, showed 100% hemolysis. The hemolysis rate of BCH-4 was considerably higher than that of BCH-1, according to the results. It could be because, during immersion, some nanofragments of rGO that were not linked to the composite hydrogel were released. These rGO nanofragments were unfriendly to blood and increased the hemolysis rate [49]. Hemolysis that is less than 5% toxic is considered nontoxic. Less than 5% hemolysis was observed in all bioactive nanocomposites. Bioactive composite hydrogels (BCH-1, BCH-2, BCH-3, and BCH-4) showed very little or no hemolysis even at high concentrations (100 μg/μL). Hence, all samples of composite hydrogels were found to be excellently hemocompatible.

#### 4.7.3. Drug Release Assay

**Figure 7:**
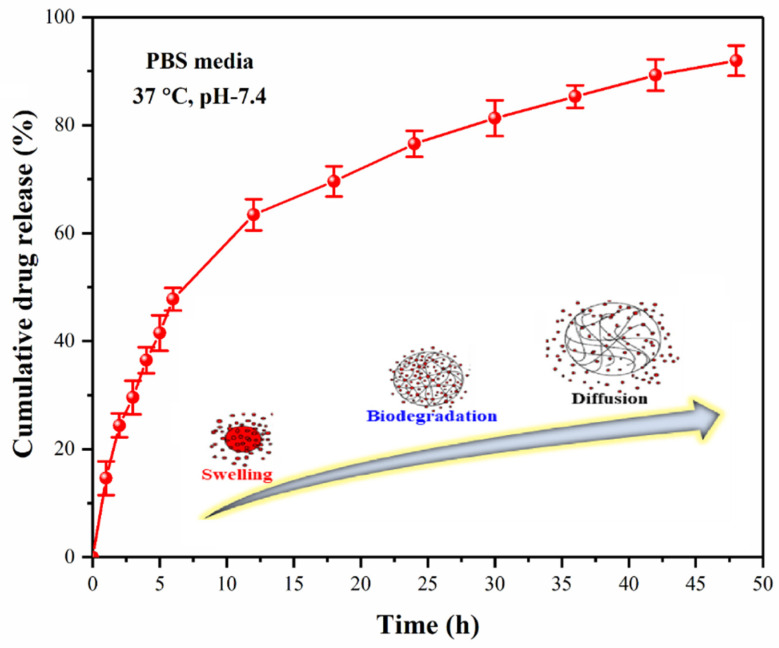
The Franz diffusion drug release profile of BCH-3 hydrogel was studied under in vitro conditions.

Silver sulfadiazine is released in a controlled manner to treat the skin wound. BCH-3 was selected for drug release analysis due to its optimized swelling, wetting, and biodegradation studies. As shown in Figure 7, silver sulfadiazine is a model antibacterial wound healing product with the controlled release (pH 7.4 at 37 °C) in PBS solution. As described by Saiful et al., in vitro drug release was determined using the Franz diffusion method. Swelling and biodegradation are essential factors in the long-term release of silver sulfadiazine at various time intervals. At 7.4 pH and 37 °C, over 60% of transdermal drug release was observed after 10 h and 90% release after 48 h. As the hydrogel film had broken into pieces, the residual amount was undetectable. To determine the behavior of drug release, we used drug release kinetic studies. As crosslinking is directly related to swelling and hydrophobicity, and changes in pH influence drug release, cross-linking also controls the long-term release of drugs [50]. As a result of their interaction with the extracellular fluid of wounds, all drug release can be controlled by controlling the hydrogels’ swelling, biodegradation, and hydrophilicity.

#### 4.7.4. Release Kinetics of Silver Sulphadiazine

**Figure 8:**
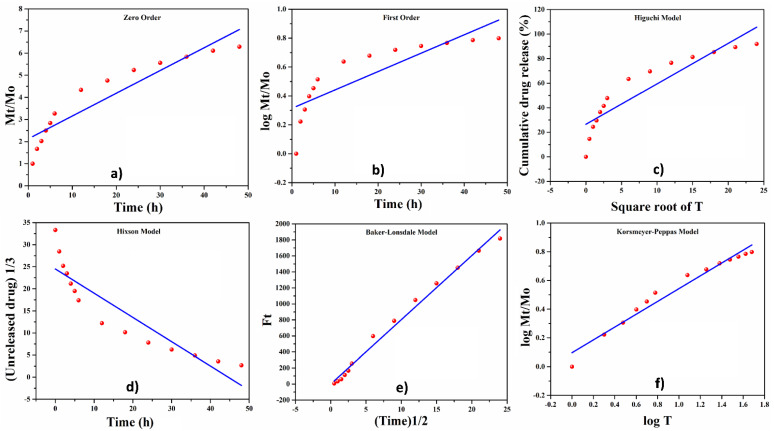
Kinetics models (**a**) zero-order, (**b**) first-order, (**c**) Higuchi, (**d**) Hixson–Crowell, (**e**) Baker–Lonsdale and (**f**) Korsmeyer–Peppas models) for drug release from hydrogels.

The drug (silver sulfadiazine) release was investigated in PBS solution via Franz diffusion to understand the release kinetic studies of silver sulfadiazine in vitro, as shown in Figure 8. As described below, we used mathematical models in Equations (5)–(10) [25,26]. Drugs are usually released from dehydrated polymeric matrices of hydrogels through a controlled swelling and diffusion mechanism involving rapid water absorption, which results in drug desorption. After water enters the glassy hydrogel matrix containing the drug, the drug is distributed into the external media via the swollen rubbery region. As regards drug swelling and diffusion, the Fickian diffusion mechanism does not apply. Instead, the non-Fickian drug release behavior is caused by the relaxation process caused by molecular electrostatic interaction during swelling and diffusion.

For the zero-order, first-order, Higuchi, Korsmeyer–Peppas, Hixson–Crowell, and Baker–Lonsdale models, the regression coefficients (R^2^) were 0.857, 0.876, 0.819, 0.963, 0.8195, and 0.989, respectively. R^2^ > 0.95 for zero-order (0.857), first-order (0.876), Higuchi (0.819), and Hixson–Crowell models was confirmed (0.8195). The regression coefficients R^2^ of Korsmeyer–Peppas (0.963) and Baker–Lonsdale (0.989) models, on the other hand, were well suited for controlled drug release, with a suitable range for drug release fitting model [24,51]. As a result, it was confirmed that Baker–Lonsdale (0.989) had the highest regression coefficient and therefore the best-fitting model among all models.

## 5. Conclusions

The composite hydrogels were prepared by a simple blending method from biopolymers for chronic wound healing. Antibacterial, pH-responsive, swelling novel hydrogels were developed and controlled the release of silver sulfadiazine via Franz diffusion. The crosslinking and hydrogen bonding of biopolymers and cross-linkers were studied and explained using FT-IR. Due to polymerization complexation, increasing the TEOS crosslinker in hydrogels increased the degradation rate and swelling but increased the water contact angle. At 37 °C, the pH response of hydrogels was observed in a variety of pH solutions. BCH-3 and BCH-4 have more potent antibacterial properties against bacteria that cause skin infections, and all samples were highly hemocompatible. All of these physicochemical properties make these hydrogels suitable biomaterials for self-wound healing. The antimicrobial and in vitro Franz diffusion-controlled release of the silver sulfadiazine drug make BCH-3 a promising biomaterial for wound care and treatment. The drug release kinetics followed the Bakers–Lonsdale model due to the higher value of the regression coefficient (R^2^ = 0.989). The physicochemical properties of hydrogels improved owing to improved cross-linking and interaction of rGO with the polymeric matrix. BCH-3 composite hydrogels are potential composite hydrogels for wound care and treatment.

## Data Availability

Data contained within the article.
